# Entry into mitosis: a solution to the decades-long enigma of MPF

**DOI:** 10.1007/s00412-015-0508-y

**Published:** 2015-02-25

**Authors:** Takeo Kishimoto

**Affiliations:** 1 Laboratory of Cell and Developmental Biology, Graduate School of Bioscience, Tokyo Institute of Technology, Yokohama, 226-8501 Japan; 2 Science and Education Center, Ochanomizu University, Ootsuka 2-1-1, Bunkyo-ku, Tokyo 112-8610 Japan

**Keywords:** Arpp19/Ensa, Cyclin B-Cdk1, Greatwall kinase, Mitosis, MPF, PP2A

## Abstract

Maturation or M phase-promoting factor (MPF) is the universal inducer of M phase common to eukaryotic cells. MPF was originally defined as a transferable activity that can induce the G2/M phase transition in recipient cells. Today, however, MPF is assumed to describe an activity that exhibits its effect in donor cells, and furthermore, MPF is consistently equated with the kinase cyclin B-Cdk1. In some conditions, however, MPF, as originally defined, is undetectable even though cyclin B-Cdk1 is fully active. For over three decades, this inconsistency has remained a long-standing puzzle. The enigma is now resolved through the elucidation that MPF, defined as an activity that exhibits its effect in recipient cells, consists of at least two separate kinases, cyclin B-Cdk1 and Greatwall (Gwl). Involvement of Gwl in MPF can be explained by its contribution to the autoregulatory activation of cyclin B-Cdk1 and by its stabilization of phosphorylations on cyclin B-Cdk1 substrates, both of which are essential when MPF induces the G2/M phase transition in recipient cells. To accomplish these tasks, Gwl helps cyclin B-Cdk1 by suppressing protein phosphatase 2A (PP2A)-B55 that counteracts cyclin B-Cdk1. MPF, as originally defined, is thus not synonymous with cyclin B-Cdk1, but is instead a system consisting of both cyclin B-Cdk1 that directs mitotic entry and Gwl that suppresses the anti-cyclin B-Cdk1 phosphatase. The current view that MPF is a synonym for cyclin B-Cdk1 in donor cells is thus imprecise; instead, MPF is best regarded as the entire pathway involved in the autoregulatory activation of cyclin B-Cdk1, with specifics depending on the experimental system.

## Introduction

**Fig. 1:**
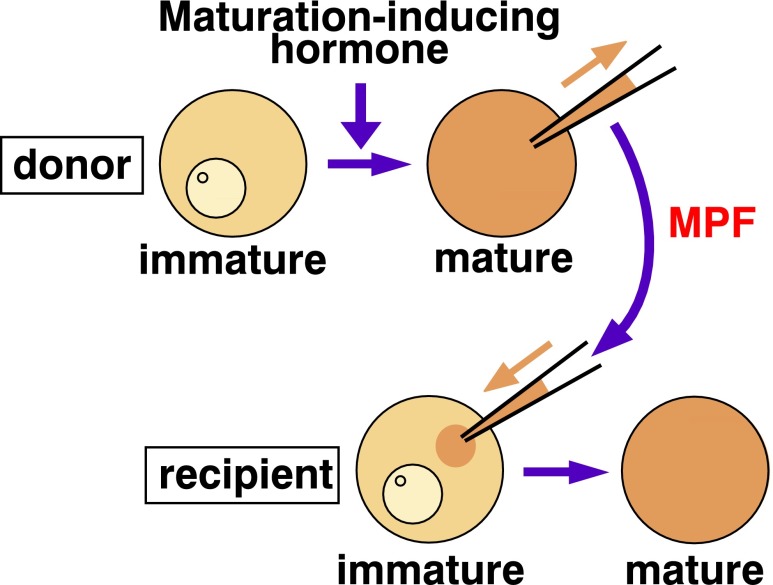
The identification of MPF through the classical microinjection assay. Maturation-promoting factor (*MPF*) was originally defined as a cytoplasmic activity transferable from mature (M phase) oocytes to immature (G2/M phase border) oocytes. MPF is present in donor oocytes at M phase, but its activity can be verified only when injected donor cytoplasm induces recipient oocytes arrested at G2 phase to transit into M phase

Today, the term maturation or M phase-promoting factor (MPF) is assumed simply to describe a molecule or molecular complex that triggers M phase within the eukaryotic cell. Originally, however, MPF was defined as a transferable activity that is not only present in the donor M phase cell but that also can induce the G2/M phase transition in the recipient G2 phase cell in the absence of new protein synthesis. MPF, designated “maturation-promoting factor”, was first demonstrated over four decades ago by Masui and Markert (1971) during investigations on oocytes and eggs of the frog *Rana pipiens*. Immature oocytes generally arrest their cell cycle at prophase of the first meiosis. Release from this arrest, which is equivalent to the G2/M phase transition in somatic cells, produces mature, fertilizable haploid eggs. Masui and Markert found a cytoplasmic activity they called MPF that is present in donor maturing (equivalent to M phase) oocytes and that upon transfer induces maturation (the G2/M phase transition) in recipient immature oocytes (Fig. 1; the classical microinjection assay of MPF).

Roughly 20 years later, the results of three separate studies on MPF, cell division cycle (*cdc*) mutants in yeasts, and cyclins in marine invertebrates suddenly converged to support the idea that MPF is the cyclin B-Cdc2 complex (reviewed by Dunphy and Newport 1988; Hunt 1989; Nurse 1990). This surprising finding enabled the “Big Bang” of highly fruitful cell cycle research during the last decade of the 20th century (during which time Cdc2 was renamed Cdk1; see Pines and Hunter 1991a; Nigg 2001). So it is not surprising that the current literature assumes that MPF is completely synonymous with cyclin B-Cdc2/Cdk1.

Nonetheless, previous studies never precisely clarified whether cyclin B-Cdk1 is, in fact, sufficient for MPF. Indeed, certain conditions have long been known in which cyclin B-Cdk1 is activated but MPF is undetectable (Kishimoto et al. 1981; Picard et al. 1988). This article will review the major gap between MPF and cyclin B-Cdk1 that exists in the historical literature and will solve this riddle by introducing another player called Greatwall kinase (Gwl; Yu et al. 2004).

## A brief history of MPF

Soon after the discovery of frog MPF (Masui and Markert 1971; Smith and Ecker 1971), similar transferable cytoplamic activity was detected in the maturing oocytes of starfish (Kishimoto and Kanatani 1976), mouse (Kishimoto et al. 1984; Sorensen et al. 1985), and other animals such as surf clam (Kishimoto et al. 1984; reviewed in Kishimoto 1988). This cytoplasmic activity was cross-reactive among different species; for example, frog MPF could induce maturation in recipient starfish oocytes (Kishimoto et al. 1982). The idea thus emerged that MPF activity is not restricted to frog oocytes alone (reviewed in Kishimoto 1988, 1996).

The general importance of MPF was further established by demonstrations that mitotically dividing cells have an equivalent activity. These MPF sources included, first, the cytoplasm of cleaving blastomeres of frog (Wasserman and Smith 1978; Gerhart et al. 1984) and starfish (Kishimoto et al. 1982); second, crude extracts from HeLa or other mammalian somatic cells synchronized at M phase (Sunkara et al. 1979; Kishimoto et al. 1982); and third, crude extracts from *cdc* mutants of the yeast *Saccharomyces cerevisiae* that were arrested in M phase by growth at the restrictive temperature (Weintraub et al. 1982; Tachibana et al. 1987). In every case, MPF activity was detected by microinjection into immature oocytes of frog or starfish. Furthermore, embryos of the frog *Xenopus laevis* that had been arrested in a G2 phase-like state by inhibition of protein synthesis underwent nuclear envelope breakdown (NEBD; a marker for M phase entry) following injection with partially purified *Xenopus* MPF (Miake-Lye et al. 1983). Although, to my knowledge, no report exists that uses somatic cells as recipients of MPF injection, experiments that were performed much earlier involving the fusion of mammalian somatic cells at different cell cycle phases (Johnson and Rao 1970) imply the presence of an activity equivalent to MPF. By the early 1980s, maturation-promoting factor (MPF) was thus envisioned to be the universal inducer of M phase in eukaryotic cells, and it was renamed M phase-promoting factor with the same abbreviation (Gerhart et al. 1985).

Beginning with Wasserman and Masui (1976) and continuing for more than a decade, many researchers tried to purify MPF, but all such attempts were unsuccessful (e.g., Wu and Gerhart 1980; Adlakha et al. 1985; Kishimoto and Kondo 1986). Finally, Maller and colleagues succeeded in purifying MPF biochemically from frog *Xenopus laevis* mature eggs by combining conventional column chromatographies with an assay system that employed cell-free egg extracts (Lohka et al. 1988). Purified frog MPF contained two major peptides of 32 and 45 kDa, and the purified preparation was associated with histone H1 kinase activity. This finding converged with great achievements in two separate fields, investigations on cell division cycle (*cdc*) mutants in yeasts and on cyclin proteins in marine invertebrates eggs, yielding the conclusion less than 2 years that MPF consists of the cyclin B-Cdc2 complex, a kinase that is typically measured by its ability to phosphorylate histone H1 (see Dunphy et al. 1988; Draetta et al. 1989; Labbe et al. 1989; Gautier et al. 1988, 1990 as representatives of many outstanding papers that led to this critical convergence of ideas; reviewed by Dunphy and Newport 1988; Hunt 1989; Nurse 1990).

## An enigma: based on the classical microinjection assay, MPF is not identical to cyclin B-Cdk1

**Fig. 2:**
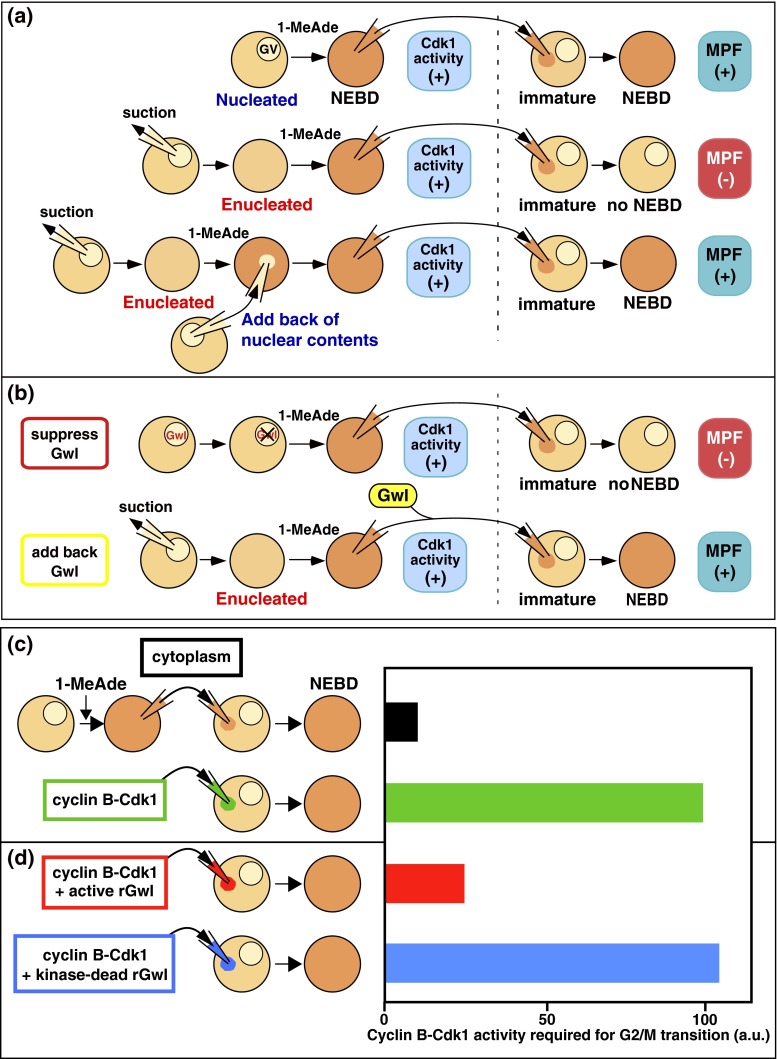
MPF is not synonymous with cyclin B-Cdk1. **a** Nuclear contents are required for MPF. In the starfish system, MPF is not detectable from enucleated donor oocytes, even those in which cyclin B-Cdk1 is activated at normal levels. But MPF is restored when nuclear contents are added back to donor enucleated oocytes. 1-Methyladenine (*1-MeAde*) is a starfish maturation-inducing hormone, which acts externally on immature oocytes to cause the G2/M phase transition. **b** Greatwall kinase (*Gwl*) is essential for MPF. When Gwl activity is suppressed in donor oocytes by injection of neutralizing antibodies, MPF is undetectable even though cyclin B-Cdk1 becomes fully activated. Conversely, Gwl restores MPF in enucleated oocytes. **c** One order of magnitude higher levels of Cdk1 activity are required for induction of NEBD in the microinjection assay, when purified cyclin B-Cdk1 is compared with cyclin B-Cdk1 contained in cytoplasmic MPF. rGwl indicates recombinant, active Gwl. **d** Addition of Gwl to purified cyclin B-Cdk1 reduces the level of Cdk1 activity required for NEBD to an amount close to that contained in cytoplasmic MPF

Certain early observations of starfish oocytes reported in the literature (Kishimoto et al. 1981; Picard and Doree 1984) were difficult to reconcile with the newly emerging view that MPF and cyclin B-Cdc2/Cdk1 were exactly the same thing. In particular, it was found that MPF is almost undetectable by cytoplasmic transfer from enucleated donor oocytes of starfish, but MPF is restored by adding back a “nuclear factor” from the germinal vesicle (GV; i.e., contents from the oocyte nucleus; see Fig. 2a; Kishimoto et al. 1981). However, cyclin B-Cdk1 is activated in enucleated donor oocytes both in terms of timing and of levels comparable to those in nucleated donor oocytes (Picard et al. 1988; see also Fig. 1 in Hara et al. 2012). These early observations clearly indicated that in the starfish oocyte system, MPF is not simply identical to cyclin B-Cdk1, but instead consists of both cyclin B-Cdk1 (found mostly in the cytoplasm; see Ookata et al. 1992) and the unknown nuclear factor (for reviews, see Kishimoto 1999; Doree and Hunt 2002).

These results with starfish contrasted markedly with findings in frog oocytes showing that MPF activity is unaffected by the presence or absence of nuclei (Masui and Markert 1971; Reynhout and Smith 1974). A likely explanation for the contrasting observations is that the starfish nuclear factor is located in the cytoplasm in the oocytes of certain frog species (see below; Hara et al. 2012).

Another discrepancy in the view that MPF = cyclin B-Cdk1 began to emerge when researchers tried to quantitate the MPF activity of purified cyclin B-Cdk1. Recall that MPF was formally defined by microinjection into immature oocytes in which its effect is verified (Fig. 1). Does purified cyclin B-Cdk1 has the same level of MPF activity in this “microinjection assay” as does donor oocyte cytoplasm contain an equivalent level of Cdk1 activity? Surprisingly, the answer was “NO” (Hara et al. 2012). In fact, purified cyclin B-Cdk1 failed to induce the G2/M phase transition in recipient oocytes of starfish, unless approximately ten-fold more activity of purified cyclin B-Cdk1 was injected than the Cdk1 activity present in the least amount of donor cytoplasm that could induce the G2/M phase transition (Okumura et al. 1996; see also Fig. 1 in Hara et al. 2012; Fig. 2c). When injected below the threshold amounts, cyclin B-Cdk1 is rapidly inactivated in the recipient starfish oocytes (Picard et al. 1991; Okumura et al. 1996). It has recently been shown that the same gap in the levels of cyclin B-Cdk1 activity required for the G2/M phase transition exists between purified kinase and donor cytoplasm in investigations of frog oocytes (Hara et al. 2012).

Thus, in addition to cyclin B-Cdk1, MPF most likely contains one or more additional components that antagonize the inactivation of this kinase. An intriguing possibility is that this antagonizing activity is carried by the nuclear factor in starfish oocytes (see below).

## MPF amplification

**Fig. 3:**
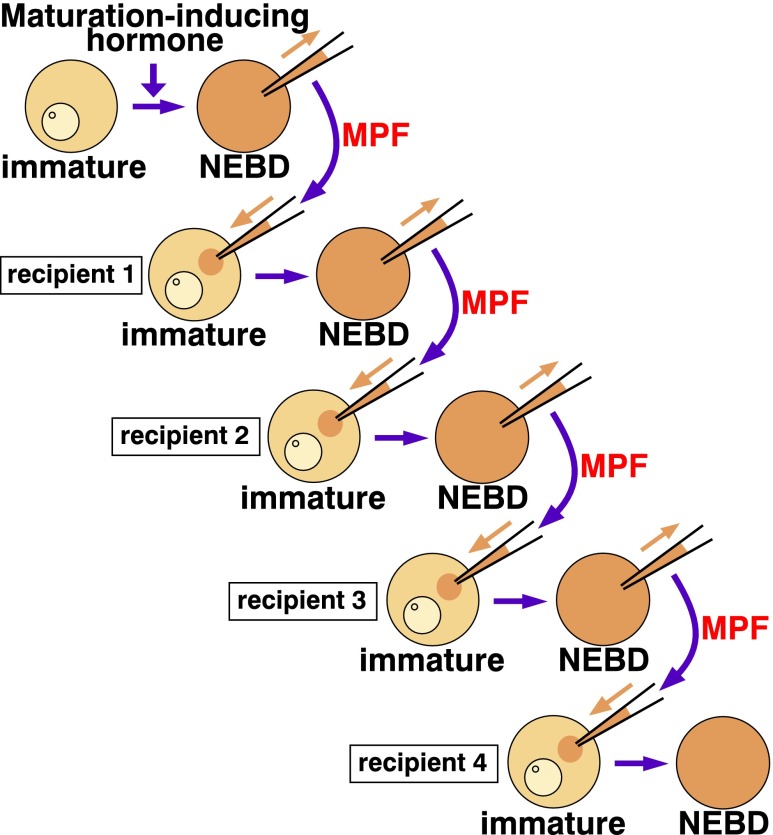
Amplification of MPF. Even after serial cytoplasmic transfer, MPF does not decrease, implying that MPF is autoactivated (“amplified”) in recipient oocytes. In the starfish system, approximately 1/15 volume of oocyte cytoplasm is transferred in each microinjection, and hence, the original MPF is diluted approximately 50,000× in the fourth recipient oocytes

**Fig. 4:**
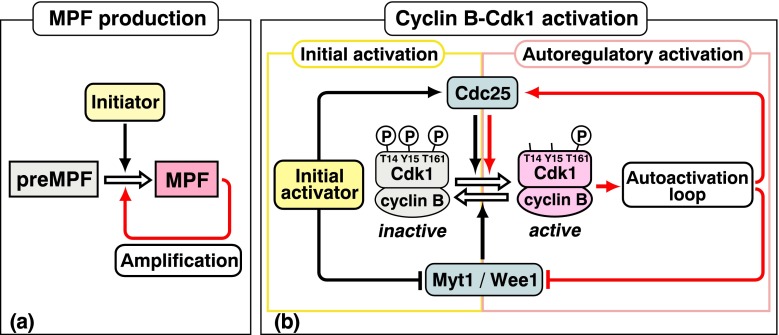
Two-step activation of MPF and cyclin B-Cdk1. **a** Initial activation and amplification of MPF. Initiator activates a small amount of preMPF to MPF (initial activation), and thereafter, MPF itself activates large amounts of preMPF to MPF (amplification). **b** Initial activation and autoregulatory activation of cyclin B-Cdk1. At the initial onset of the G2/M phase transition, a putative initial activator reverses the balance between Cdc25 and Myt1/Wee1 to trigger activation of a small amount of cyclin B-Cdk1 (initial activation), and thereafter, the active cyclin B-Cdk1 starts the autoactivation loop to induce activation of large amounts of cyclin B-Cdk1 (autoregulatory activation)

In retrospect, the first frog MPF article by Masui and Markert (1971) contained an important clue for a possible role of the nuclear factor. The authors found that MPF has an autocatalytic property called “amplification,” based on the observation that the MPF activity contained in the cytoplasm does not decrease through multiple successive transfers into immature oocytes (Fig. 3). Such MPF amplification was observed in starfish oocytes as well (Kishimoto and Kanatani 1976). The amplification of MPF occurs normally even when protein synthesis is suppressed in the oocytes of frog (Wasserman and Masui 1975) and starfish (Doree 1982). These observations taken together suggest that a precursor of MPF (preMPF) is present in immature oocytes and can be transformed into MPF through the action of MPF itself. Accordingly, the process of MPF production in oocytes can be divided into two steps (Fig. 4a). First, some kind of “initiator”—a signal independent of MPF—allows the production of a small amount of MPF (i.e., “primary MPF”) from preMPF. Thereafter, the autoregulatory amplification process allows the production from preMPF of large amounts of MPF (“amplified MPF”); this amplification depends on MPF itself. During the microinjection assays described above, the MPF introduced into recipient oocytes functions as the primary MPF; it starts the MPF amplification loop in recipient oocytes despite the absence of the initiator.

Is the nuclear factor involved in the production of primary MPF or in the production of amplified MPF? If the first recipient oocytes have been enucleated in the serial transfer experiment using starfish oocytes (Fig. 3), no NEBD occurs in the second recipient oocytes (Kishimoto et al. 1981), even though cyclin B-Cdk1 is fully activated in the enucleated first recipient oocytes (Picard et al. 1991). These observations indicate that the nuclear factor of donor oocytes is required for the MPF amplification in recipient oocytes. A likely explanation for this requirement is that in the absence of the nuclear factor, small amounts of primary MPF in the recipient are inactivated before they can initiate the amplification process.

## Two-step activation of cyclin B-Cdk1

After the cyclin B-Cdk1 complex is first formed, its activity is directly regulated negatively by Wee1/Myt1 kinase that phosphorylates Cdk1 for inhibition and positively by Cdc25 phosphatase that dephosphorylates the Wee1/Myt1 sites for activation (for review, see Lew and Kornbluth 1996; Fig. 4b). At the G2/M phase border, all of the cyclin B-Cdk1 complex, Wee1/Myt1 and Cdc25 proteins are already present, but cyclin B-Cdk1 is kept inactive, implying that the balance of activity between Wee1/Myt1 kinase and Cdc25 phosphatase is inclined to the inhibitory phosphorylation of Cdk1.

At the onset of M phase, the balance between Wee1/Myt1 and Cdc25 is first reversed by cyclin B-Cdk1-independent, upstream signaling (the putative “initial activator”), resulting in a small population of active cyclin B-Cdk1. Subsequently, a much larger population of cyclin B-Cdk1 becomes activated through an autoregulatory loop in which active cyclin B-Cdk1 further inactivates Wee1/Myt1 and activates Cdc25 (for reviews, see Lew and Kornbluth 1996; O’Farrell 2001; Ferrell et al. 2009; Lindqvist et al. 2009; Santos et al. 2012; Fig. 4b). The initial activation of cyclin B-Cdk1 may roughly (but not precisely) correlate with production of the primary MPF and the autoregulatory activation of cyclin B-Cdk1 with the MPF amplification.

### Initial activation of cyclin B-Cdk1

Logically, prior to starting the autoactivation loop, a small amount of cyclin B-Cdk1 should be activated by the cyclin B-Cdk1-independent, putative initial activator (Fig. 4b). It remains unclear in vivo in most higher eukaryotic somatic cells what molecule(s) is(are) the actual initial activator(s) of cyclin B-Cdk1, although Aurora A, Plk1, cyclin A-Cdk1/2, and Cdc25B might all be involved in tipping the balance between Wee1/Myt1 and Cdc25 (for reviews, see Nigg 2001; O’Farrell 2001; Lindqvist et al. 2009). Some investigators regard the initial activation to be the result of redundant or stochastic processes involving these molecules (O’Farrell 2001; Lindqvist et al. 2009).

**Fig. 5:**
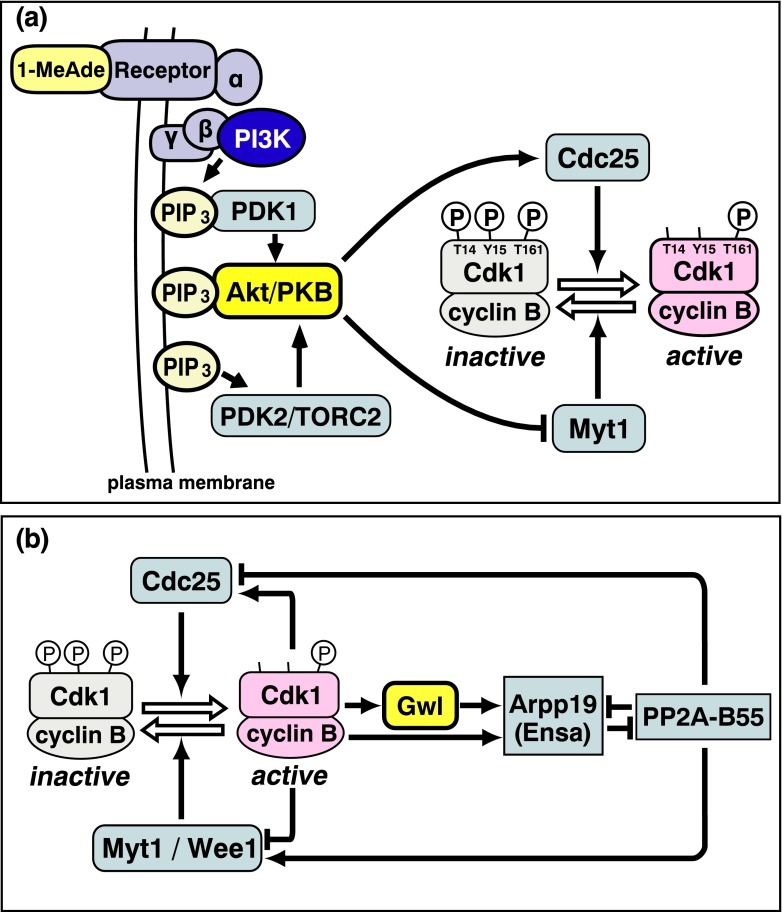
Pathways leading to initial activation and autoregulatory activation of cyclin B-Cdk1. **a** Pathway toward the initial activation of cyclin B-Cdk1 in starfish oocytes. A putative receptor of maturation-inducing hormone (1-MeAde) is localized on the oocyte surface. Downstream of this signal, Akt/PKB is activated and then directly phosphorylates both Cdc25 and Myt1 to reverse their activity balance, triggering activation of cyclin B-Cdk1. Akt/PKB thus clearly functions as the initial activator. **b** The cyclin B-Cdk1 autoactivation loop. A key element is Arpp19/Ensa-dependent inhibition of PP2A-B55, which counteracts phosphorylations of Cdc25 and Myt1/Wee1 by cyclin B-Cdk1. Activation of Arpp19/Ensa is accomplished through phosphorylation either by cyclin B-Cdk1 itself or by Gwl that is activated downstream of cyclin B-Cdk1

In contrast, the starfish oocyte represents an exceptional system in which the initial activator is well characterized (Fig. 5a). Akt/PKB, which is activated downstream of the starfish maturation-inducing hormone (1-methyladenine, 1-MeAde; Kanatani et al. 1969) with no requirement of new protein synthesis, clearly functions as an in vivo initial activator (Okumura et al. 2002). Akt/PKB directly phosphorylates both Cdc25 and Myt1 to reverse the balance of their activities, resulting in net removal of inhibitory phosphorylations on Cdk1 (for reviews, see Kishimoto 2003, 2011). As in most animal species, Wee1 is absent from immature oocytes of starfish (Okano-Uchida et al. 2003); furthermore, activation of Aurora (Abe et al. 2010) and Plk1 (Okano-Uchida et al. 2003) and new synthesis of cyclin A and Mos (Tachibana et al. 2000) are absolutely downstream of, and not required for, cyclin B-Cdk1 activation in starfish.

In *Xenopus* oocytes, progesterone downregulates cAMP-dependent protein kinase A (PKA). Downstream of PKA inactivation, new protein synthesis either of Mos or cyclin B is required to trigger the initial activation of pre-existing cyclin B-Cdk1 through the removal of the inhibitory phosphorylation on Cdk1 (Haccard and Jessus 2006a). However, the biochemical steps that link PKA downregulation to the protein synthesis of these intermediaries remain unclear (Haccard and Jessus 2006b).

### Autoregulatory activation of cyclin B-Cdk1

Core elements of the autoregulatory loop are cyclin B-Cdk1-dependent phosphorylation both of Cdc25 for further activation and of Myt1/Wee1 for further inactivation. Although many of the phosphorylations of Cdc25 and Myt1/Wee1 responsible for the positive feedback are directly catalyzed by cyclin B-Cdk1 (O’Farrell 2001; Lindqvist et al. 2009), the autoregulatory loop is not so simple because it also includes antagonizing action against the protein phosphatase that counteracts phosphorylations by cyclin B-Cdk1 (Fig. 5b). Emerging studies indicate that M phase is controlled by highly coordinated activities between multiple protein kinases and opposing protein phosphatases (for reviews, see Lindqvist et al. 2009; Mochida and Hunt 2012; Qian et al. 2013). Typically, many of the cyclin B-Cdk1-catalyzed phosphorylations of Cdc25 and Myt1/Wee1 are in large part opposed by heterotrimeric PP2A that contains a regulatory subunit of the B55 family (PP2A-B55; Mochida et al. 2009). Accordingly, a key issue in understanding the autoregulatory activation is how cyclin B-Cdk1 turns off PP2A-B55.

Mutations in the gene encoding Greatwall kinase (Gwl) in the fruit fly *Drosophila* were first identified as a dominant allele called *Scott of the Antarctic* (*Scant*; White-Cooper et al. 1996) and as recessive alleles called *greatwall* (*gwl*; Yu et al. 2004). Gwl itself was originally described as a nuclear protein required for proper chromosome condensation and M phase progression (Yu et al. 2004; Archambault et al. 2007). Further studies in frog *Xenopus* eggs and their extracts revealed several points that are illustrated in Fig. 5b. (1) Cyclin B-Cdk1 is important for Gwl activation (Yu et al. 2006; Vigneron et al. 2011; Blake-Hodek et al. 2012). (2) Gwl participates in the autoactivation loop of cyclin B-Cdk1 (Yu et al. 2006). (3) This action of Gwl in the autoactivation loop includes negative regulation of PP2A-B55 (Zhao et al. 2008; Vigneron et al. 2009; Castilho et al. 2009). (4) This suppression is accomplished through direct phosphorylation by Gwl of Ensa/Arpp19 (Ensa, α-endosulfine; and its close relative Arpp19, cyclic adenosine monophosphate-regulated phosphoprotein 19) that, in turn, leads to inhibition of PP2A-B55 (Gharbi-Ayachi et al. 2010; Mochida et al. 2010). (5) The phosphorylation and activation of Ensa/Arpp19 by Gwl are opposed by PP2A-B55 (Williams et al. 2014). At least in the frog egg system, the cyclin B-Cdk1-Gwl-Ensa/Arpp19 pathway is critical for suppressing PP2A-B55 that counteracts cyclin B-Cdk1 (Fig. 5b). In this way, cyclin B-Cdk1-driven phosphorylations of Cdc25 and Myt1/Wee1 can be maintained to promote the autoregulatory activation of cyclin B-Cdk1.

In mammalian somatic cells, however, Gwl/MASTL (the mammalian ortholog of Gwl) is largely dispensable for entry into M phase, though it is essential for M phase progression (Burgess et al. 2010; Voets and Wolthuis 2010; Alvarez-Fernandez et al. 2013; Cundell et al. 2013). Gwl-null cells enter into M phase with normal kinetics; after NEBD, they display features of mitotic collapse such as defective condensation and segregation of chromosomes, prometaphase arrest, and disordered cytokinesis. The fact that such cells can enter M phase suggests a possible autoregulatory activation of cyclin B-Cdk1 in the absence of Gwl, although the degree of activation may be suboptimal. Among invertebrates, the nematode *Caenorhabditis elegans* has no obvious Gwl in its genome (Kim et al. 2012), whereas cyclin B-Cdk1 is believed to function as in other eukaryotic cells. Most strikingly, although the starfish oocyte indeed has Gwl that is exclusively present in the germinal vesicle (GV; i.e., the oocyte nucleus), cyclin B-Cdk1 can be nonetheless fully activated both in enucleated oocytes that lack Gwl and in nucleated oocytes in which Gwl activity is suppressed by antibody injection (Hara et al. 2012; see Fig. 2a, b). All of these facts support the idea that the autoregulatory activation of cyclin B-Cdk1 can be accomplished even in the absence of Gwl.

How is it possible that cyclin B-Cdk1 can be activated if Gwl is not present? The probable answer involves two facts which were found in the starfish oocyte system (Okumura et al. 2014). First, what is essential for the autoactivation loop is not Gwl but is instead the downstream Arpp19 (and most likely Ensa); and second, cyclin B-Cdk1 directly phosphorylates Arpp19 on a conserved site (Ser69) different from that targeted by Gwl, resulting in inhibition of PP2A-B55. Although Arpp19 phosphorylated by cyclin B-Cdk1 alone is significantly less effective in inhibiting PP2A-B55 than Arpp19 phosphorylated by cyclin B-Cdk1 plus Gwl, a partial reduction of PP2A-B55 activity by the cyclin B-Cdk1-Arpp19 bypass is most likely sufficient for the autoregulatory activation of cyclin B-Cdk1 (Fig. 5b).

The homologous site for Ser69 of starfish Arpp19 is present in human Arpp19 on Ser23, on Ser28 of frog Arpp19, on Thr28 of frog Ensa, and on Ser21 of *C. elegans* Ensa (Okumura et al. 2014). This site is not present in human Ensa or in the single Ensa family member in *Drosophila*, although, in fruit flies, the equivalent position is a phosphomimetic aspartic acid (Kim et al. 2012). Given that the homologous site is present in both frog Ensa and frog Arpp19, it is somewhat puzzling why Gwl is essential for cyclin B-Cdk1 activation in cycling extracts from frog eggs (Yu et al. 2006). Possible explanations for the frog case might involve the unusual cytoplasmic localization of Gwl in frog oocytes (Hara et al. 2012) and/or the use of self-oscillatory egg extracts whose autoregulation may vary slightly from that seen in vivo. Taken together, certainly in several systems, it appears that direct phosphorylation by cyclin B-Cdk1 or a phosphomimetic mutation of at least one Arpp19/Ensa family member is sufficient for at least a partial autoregulatory activation of cyclin B-Cdk1.

These many observations can be rationalized by a simple model. In normal cells containing Gwl, the cyclin B-Cdk1-Arpp19/Ensa bypass might start the autoactivation loop immediately after the initial activation of cyclin B-Cdk1. Subsequently, after cyclin B-Cdk1 activates Gwl, both the cyclin B-Cdk1-Arpp19/Ensa bypass and the cyclin B-Cdk1-Gwl-Arpp19/Ensa pathway would act synergistically to accomplish the swift and robust autoactivation of cyclin B-Cdk1 (Fig. 5b).

## MPF needs to antagonize the reactions that oppose cyclin B-Cdk1

At the G2/M phase border, activation of cyclin B-Cdk1 is strictly prevented by Myt1/Wee1 and PP2A-B55, while activation of Cdc25 coupled with inactivation of Myt1/Wee1 ensure the swift and robust activation of cyclin B-Cdk1 at entry into M phase. Under physiological conditions, both Cdc25 activation and Myt1/Wee1 inactivation are accomplished first by the initial activator and, subsequently, via the autoregulatory loop (see above; Fig. 4b), implying that the autoregulatory activation of cyclin B-Cdk1 is initiated after the balance between Myt1/Wee1 and Cdc25 has been first reversed by the initial activator (see Fig. 3 in Okumura et al. 1996; Fig. 1 in Okumura et al. 2002). But in the microinjection assay, MPF from the donor oocyte is forced to initiate the autoregulatory activation of cyclin B-Cdk1 in the recipient oocyte without the aid of the initial activator and thus in the complete absence of both Cdc25 activation and Myt1/Wee1 inactivation. This fact implies that the active cyclin B-Cdk1 contained in donor MPF would be opposed by two major reactions in recipient cells. First, the introduced cyclin B-Cdk1 would be directly attacked by Myt1/Wee1. Second, even if this cyclin B-Cdk1 could survive this first attack, its ability to start the cyclin B-Cdk1 autoactivation loop would then be opposed by PP2A-B55.

These two opposing reactions against the active donor cyclin B-Cdk1 that is introduced into G2 phase cells could explain why MPF requires the nuclear factor in addition to cyclin B-Cdk1 in starfish; that is, the nuclear factor might antagonize the opposing reactions through supporting the autoregulatory activation of cyclin B-Cdk1. Intriguingly, Picard et al. (1991) found that okadaic acid, a potent inhibitor of PP2A, potentiates the ability of a low, physiological level of purified cyclin B-Cdk1 to induce the G2/M phase transition in the microinjection assay (i.e., this level of cyclin B-Cdk1 is insufficient to induce the transition if okadaic acid is not present) . If this potentiation is executed through the autoregulatory activation of cyclin B-Cdk1, PP2A inhibition could be involved in MPF amplification. Based on these considerations, the authors proposed that the nuclear factor might be a PP2A inhibitor which acts synergistically with cyclin B-Cdk1 to induce MPF amplification.

These findings, taken together with recent knowledge about the suppression of PP2A-B55 activity during M phase, suggest the plausible hypothesis that the nuclear factor required for MPF could be a component of the pathway that leads to activation of Arpp19/Ensa for PP2A inhibition.

## The enigma’s solution: Gwl and cyclin B-Cdk1 together constitute MPF

Which component of the cyclin B-Cdk1-Gwl-Arpp19/Ensa pathway or the cyclin B-Cdk1-Arpp19/Ensa bypass (Fig. 5b) is localized exclusively in the nucleus? Cyclin B-Cdk1 is localized in the cytoplasm at the G2/M phase border (Pines and Hunter 1991b; Ookata et al. 1992), and PP2A-B55 is largely cytoplasmic (Mayer-Jaekel et al. 1994; Santos et al. 2012; Alvares-Fernandez et al. 2013). Ensa is present in both the cytoplasm and the nucleus in the fruit fly (Rangone et al. 2011), while Arpp19 is largely cytoplasmic in starfish oocytes (Okumura et al. 2014), excluding these molecules as candidates for the nuclear factor. In contrast, Gwl was originally described as a nuclear protein (Yu et al. 2004; see also Wang et al. 2013), supporting Gwl as the most likely candidate for the nuclear factor.

**Fig. 6:**
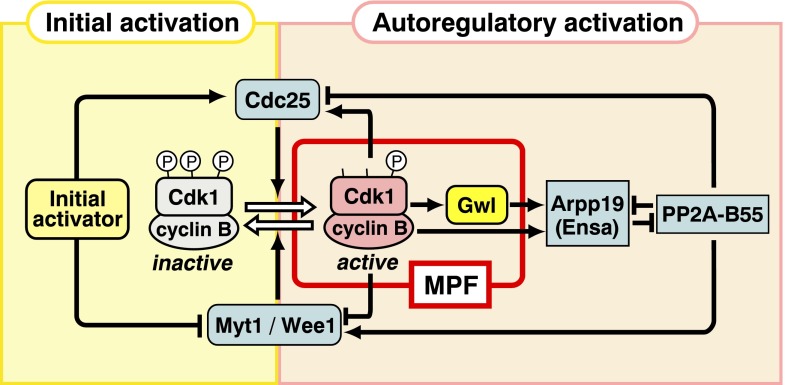
MPF and cyclin B-Cdk1 activation. In vivo, cyclin B-Cdk1 activation is accomplished via the initial activation step and the following autoregulatory activation step. In contrast, MPF, which is introduced into recipient cells, skips the initial activation step and is forced to start the autoactivation loop to accomplish the activation of cyclin B-Cdk1. In the absence of the initial activation step, the balance in the cell of Cdc25 and Myt1/Wee1 activities is weighted toward the inactivation of cyclin B-Cdk1. This is the reason why a physiological level of cyclin B-Cdk1 alone is insufficient for MPF and why Gwl is further required for MPF. Based on the classical microinjection assay, MPF thus consists of both cyclin B-Cdk1 and Gwl

Gwl is indeed localized exclusively in the nucleus (GV) in immature starfish oocytes as well, and it is activated immediately after, and downstream of, the activation of cyclin B-Cdk1 during the G2/M phase transition (Hara et al. 2012). When Gwl activity is suppressed through antibody injection, MPF is undetectable even from nucleated oocytes in which cyclin B-Cdk1 is fully activated. Conversely, MPF is restored when Gwl is added back to enucleated donor oocytes or when Gwl is supplemented to the cytoplasm obtained from enucleated donor oocytes (Fig. 2b), whereas Gwl alone fails to exhibit MPF function even when added at seven-fold excess of the physiological amounts. Furthermore, addition of recombinant Gwl to purified cyclin B-Cdk1 greatly reduces the amount of cyclin B-Cdk1 required for the microinjection assay of MPF (Fig. 2d). This great reduction is the case in *Xenopus* oocytes as well. Although cyclin B-Cdk1 alone can induce NEBD when injected at ten-fold excess the physiological amounts, spindle assembly thereafter is abortive; in contrast, when accompanied by Gwl, normal amounts of cyclin B-Cdk1 lead to formation of apparently normal spindles. These results allow us to conclude that Gwl itself is the essential nuclear factor required for MPF (Hara et al. 2012; Fig. 6).

Although Gwl was identified as an essential component of classically defined MPF in the oocyte, one specialized type of germline cell, Gwl may be a critical constituent of MPF in the somatic cells of higher eukaryotes as well, because Gwl/MASTL is also nuclear in human somatic cells (Burgess et al. 2010; Alvarez-Fernandez et al. 2013) and in *Drosophila* cells (Yu et al. 2004; Wang et al. 2013). This supposition might explain why the literature has no reports that the simple introduction of active cyclin B-Cdk1 causes G2/M phase transition in somatic cells (Hagting et al. 1998; Fung et al. 2007).

Given that Arpp19/Ensa can be directly activated by cyclin B-Cdk1 in the bypass pathway (Okumura et al. 2014), Arpp19 should be active in hormone-treated enucleated starfish oocytes, but MPF is undetectable from these oocytes (Kishimoto et al. 1981; Picard and Doree 1984). Why can’t the cyclin B-Cdk1-activated Arpp19/Ensa replace Gwl as a component of MPF? According to the recent “unfair competition model” proposed by Williams et al. (2014), the Gwl phosphorylation of Arpp19/Ensa is reversed by PP2A-B55 itself. In contrast, the cyclin B-Cdk1-mediated phosphorylation of Arpp19/Ensa is very hard to remove at M phase exit and is reversed by unknown, okadaic acid-insensitive phosphatase other than PP2A-B55 (Williams and Goldberg, personal communication; see also Fig. S3 in Cundell et al. 2013 and Fig. 1 in Mochida 2014). It is thus less likely that the active, cyclin B-Cdk1-phosphorylated form of Arpp19/Ensa is rapidly dephosphorylated and inactivated by phosphatases in the recipient oocytes. Instead, it should be remembered that Arpp19/Ensa phosphorylated by cyclin B-Cdk1 is less efficient in suppressing PP2A-B55 than are the same proteins phosphorylated by Gwl (Mochida 2014; Okumura et al. 2014); and further that the active Arpp19/Ensa is diluted approximately 15-fold after microinjection into the recipient oocytes (see Fig. 3 legend). These may make the introduced Arpp19/Ensa inefficient to inhibit PP2A-B55 in the recipient oocytes. In short, the cyclin B-Cdk1-phosphorylated form of Arpp19/Ensa alone is insufficient: The coexistence of active Gwl kinase is needed to maintain the suppression of PP2A-B55 so as to start the autoactivation loop for cyclin B-Cdk1 in recipient cells.

Based on its classical definition through microinjection assay, MPF is thus not synonymous with cyclin B-Cdk1, but instead, MPF is composed of both cyclin B-Cdk1 and Gwl. In other words, MPF is a system consisting of both cyclin B-Cdk1 that directs mitotic entry and Gwl that suppresses the anti-cyclin B-Cdk1 phosphatase, PP2A-B55.

## Other historical riddles about MPF

### Why is MPF detectable from enucleated frog oocytes?

It is well known that enucleation does not prevent the appearance of MPF in oocytes of the frog species *R. pipiens* (Masui and Markert 1971) and *X. laevis* (Iwashita et al. 1998). Recall that immature frog oocytes are exceptional in that Gwl is mostly localized in their cytoplasm (Hara et al. 2012). This localization explains why MPF is detectable from enucleated *Xenopus* oocytes that would contain both active cyclin B-Cdk1 and active Gwl. Even so, evidence exists that in the *Xenopus* oocyte system as well, Gwl contributes to MPF through reducing the required amount of cyclin B-Cdk1 (Hara et al. 2012), suggesting that the contribution of Gwl to MPF is most likely a general feature regardless of Gwl’s intracellular localization. A plausible explanation for the biological implications of species differences in the nuclear versus cytoplasmic localization of Gwl would be that the unusually large cytoplasmic volume of the frog oocyte may make nuclear localized Gwl inadequate to support the swift and robust autoactivation of cyclin B-Cdk1.

### Timing of MPF detection in oocytes

In its original definitions, MPF was regarded to be a cytoplasmic activity that controls the nucleus (Masui and Markert 1971) or that mediates transfer of the maturation-inducing hormonal stimulus from the oocyte surface to the nucleus (Kishimoto and Kanatani 1976). If so, in the classical microinjection assay, MPF should be principally detectable within the cytoplasm of donor oocytes prior to NEBD. MPF in *Rana* oocytes behaves exactly in this way (Masui and Markert 1971), and we can now explain this fact by the cytoplasmic localization of Gwl in frog oocytes (Hara et al. 2012). In contrast, starfish MPF is very hard to detect until just before the occurrence of NEBD (Kishimoto and Kanatani 1976), despite the facts that cyclin B-Cdk1 is already almost fully activated before NEBD (Ookata et al. 1992) and that Gwl is fully activated very soon after cyclin B-Cdk1 activation (Hara et al. 2012). Instead, prior to NEBD in hormone-treated oocytes, MPF is occasionally detectable in the nucleus but not in the cytoplasm (Picard and Doree 1984; Picard et al. 1991).

Although these observations have long been puzzling, we can now understand why MPF from starfish behaves as it does. First, after it is activated in the cytoplasm, cyclin B-Cdk1 is transported into the nucleus and accumulates there (Ookata et al. 1992). Second, Gwl is exclusively localized in the nucleus until just before NEBD (Hara et al. 2012), so Gwl activation most likely occurs in the nucleus only after active cyclin B-Cdk1 has been imported. Third, Gwl is likely to be exported from the nucleus to the cytoplasm just before NEBD, as has recently been shown in somatic cells (Wang et al. 2013; Alvarez-Fernandez et al. 2013). These points account for the previously confusing behavior of MPF in starfish, explaining both why MPF detection in starfish oocytes is easier after NEBD than before NEBD, and why MPF is detectable in the nucleus but not in the cytoplasm before NEBD.

## Concluding remarks: reconciling MPF, Gwl, and cyclin B-Cdk1

After four decades, it is now finally clear that according to the classical microinjection assay, MPF consists not only of cyclin B-Cdk1 but also of Gwl. It required 20 years for the identification of cyclin B-Cdk1 and another 20 years for the identification of Gwl. Unfortunately, however, a major gap now exists between the relatively imprecise way in which the current literature uses the term “MPF” as a synonym for cyclin B-Cdk1 and the original functional definition of MPF, which now must encompass Gwl as well.

How can this gap be reconciled? The reason this gap exists involves two features of the classical MPF assayed functionally by microinjection: This MPF activity is transferable, and it can be amplified. All of the components of MPF are derived from donor cells, but the presence of classical MPF can be verified only by its activity in recipient cells. In contrast, when researchers use the term MPF today as a synonym for cyclin B-Cdk1 (“current MPF” below), they are describing an activity that exists in donor cells and is verified biochemically as M phase-specific histone H1 kinase. In vivo in donor cells, the activation of cyclin B-Cdk1 requires that the balance between Cdc25 and Myt1/Wee1 must first be tipped by the initial activator, and thereafter, the small resultant amount of active cyclin B-Cdk1 starts its autoactivation loop (Fig. 6). In contrast, when the classical MPF is transferred and induces the G2/M phase transition in recipient cells, the introduced active cyclin B-Cdk1 must start its autoactivation loop under circumstances in which the initial activator-dependent reversal of the balance between Cdc25 and Myt1/Wee1 has not yet been accomplished. This skipping of the initial activator-dependent step explains why classically defined MPF requires Gwl in addition to cyclin B-Cdk1. In other words, when the initial activation step is already accomplished, a low level of cyclin B-Cdk1 alone is sufficient to drive the autoactivation loop (a situation seen in the current MPF); but when the initial activation step is bypassed, the autoactivation loop requires either the addition of Gwl to low levels of cyclin B-Cdk1 (reconstituting the classical MPF) or an extraordinarily high activity level of purified cyclin B-Cdk1 alone (Okumura et al. 1996; Hara et al. 2012).

The gap between the classical MPF and the current MPF thus reflects differences in the assay systems employed and quantification of cyclin B-Cdk1 activity. Considering that MPF was originally identified as the cytoplasmic activity, it is misleading to suggest that there is one universal MPF molecule(s). Instead, MPF is most accurately regarded as a pathway whose specifics depend on the experimental system being investigated. In other words, we should now understand that MPF in its most fundamental sense is not only cyclin B-Cdk1, but is instead a complex network involved in the autoregulatory activation of cyclin B-Cdk1.

The finding that cyclin B-Cdk1 phosphorylation of Arpp19/Ensa is sufficient to drive the cyclin B-Cdk1 autoactivation loop in the absence of Gwl (Okumura et al. 2014) raises the question: What essential role does Gwl play? In fact, in many systems including human somatic cells, Gwl exhibits more impact after NEBD: Gwl is nonessential for mitotic entry, but it is necessary for further mitotic progression including events such as chromosome condensation (Alvarez-Fernandez et al. 2013), the proper segregation of chromosomes (Okumura et al. 2014), and orderly cytokinesis following chromosome separation (Cundell et al. 2013). One intriguing possibility would be that Gwl regulates these post-NEBD events by phosphorylating other currently unknown substrates besides Arpp19/Ensa. An alternative possibility is that Arpp19/Ensa inhibits PP2A-B55 in a biphasic manner (Okumura et al. 2014). That is, more potent Arpp19/Ensa phosphorylated by both cyclin B-Cdk1 and Gwl might be necessary for these events that occur after cyclin B-Cdk1 activation, whereas less potent Arp19/Ensa phosphorylated by cyclin B-Cdk1 alone is sufficient for cyclin B-Cdk1 autoactivation, but is not sufficient for these later events. A plausible basis for this hypothesis is that various phosphoprotein substrates have different sensitivities to various levels of PP2A-B55 phosphatase activity: It will therefore be intriguing to identify the substrates that exhibit greater or lesser sensitivities to the inhibition of PP2A-B55.
